# Supplementary figures and images for: Advancing gender equality in global health: What can we learn from successful gender integration across five UN agencies?
Source: PLOS Glob Public Health. 2025 Jun 4;5(6):e0004697. doi: 10.1371/journal.pgph.0004697 (PMC12136305; doi:10.1371/journal.pgph.0004697)

S1 Text: Summary of the 14 successful case studies


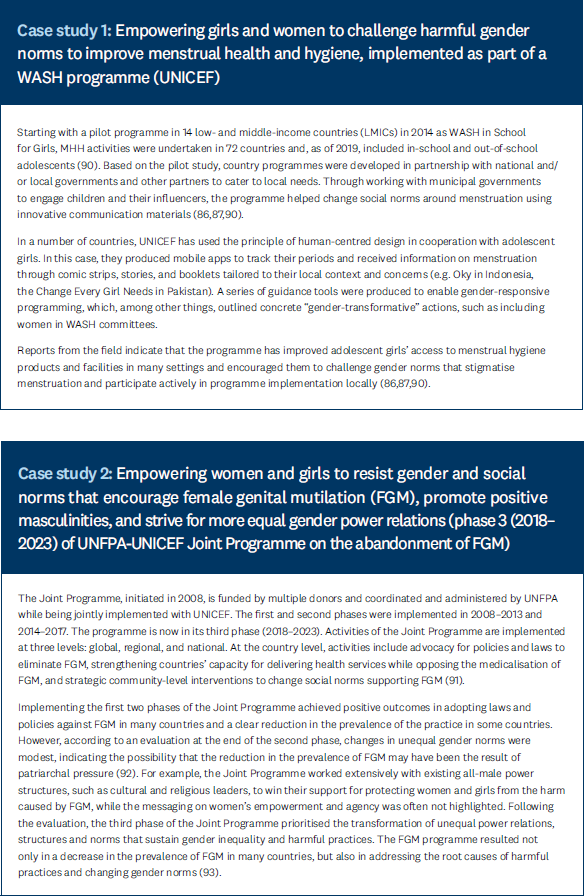


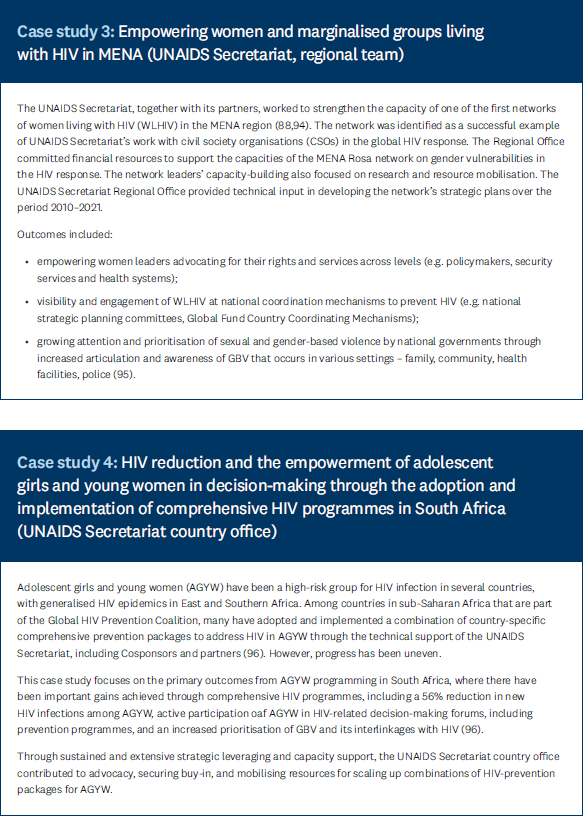


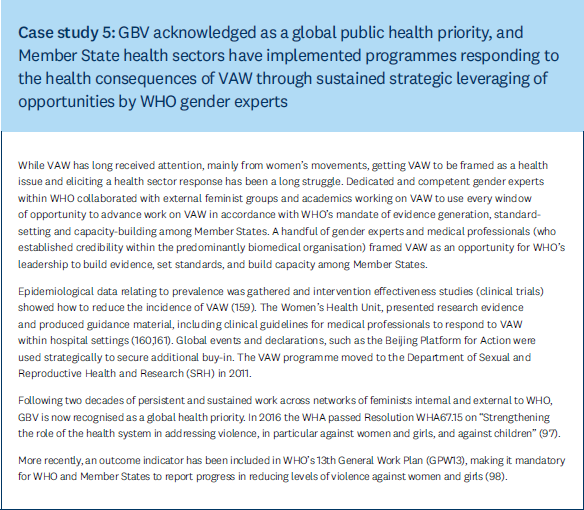


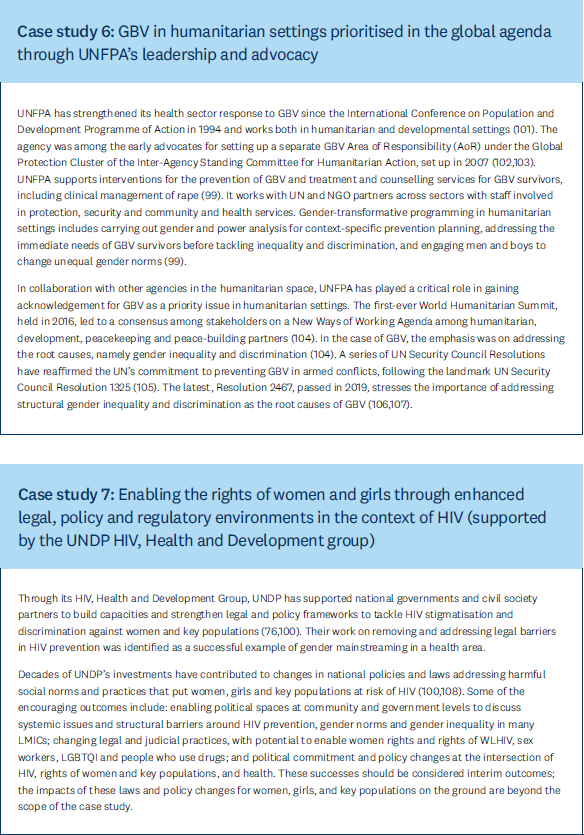


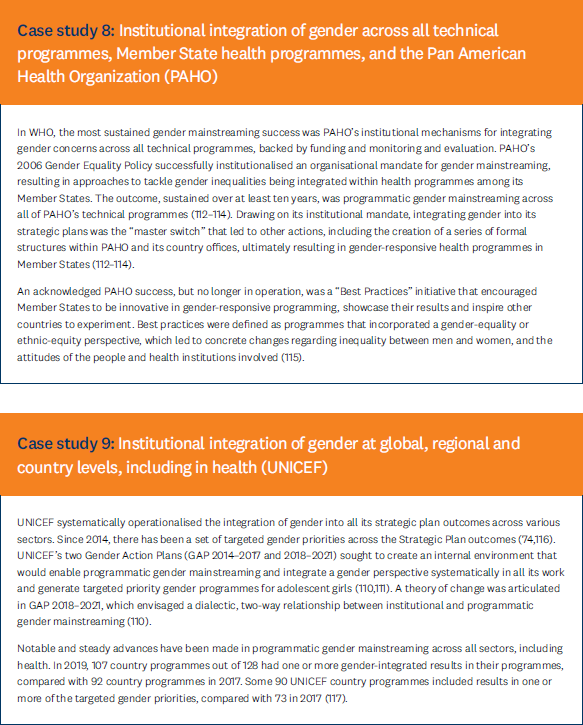


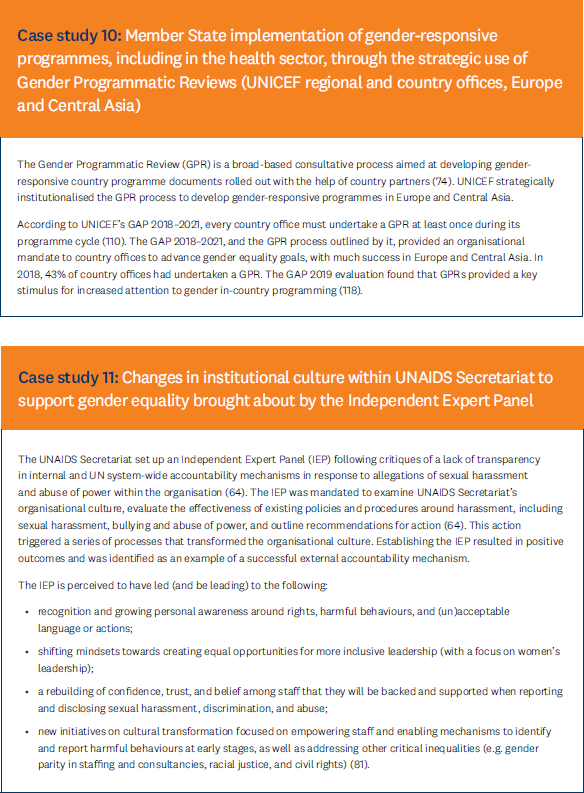


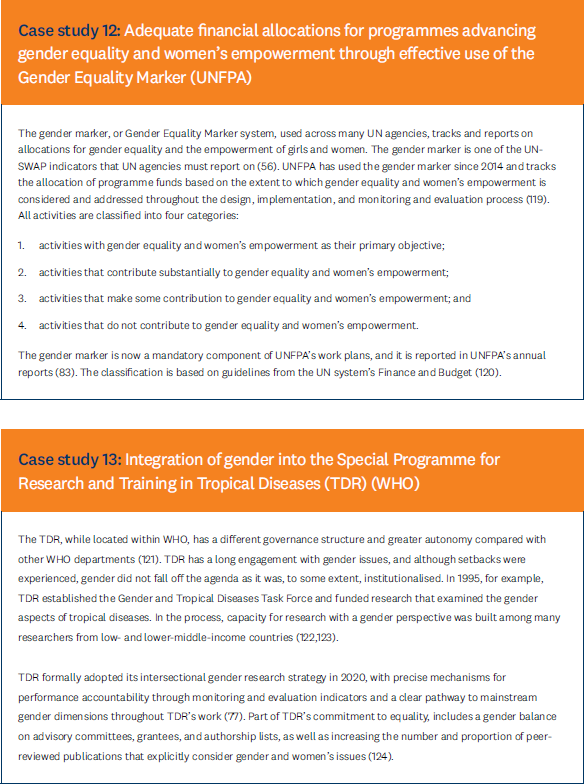


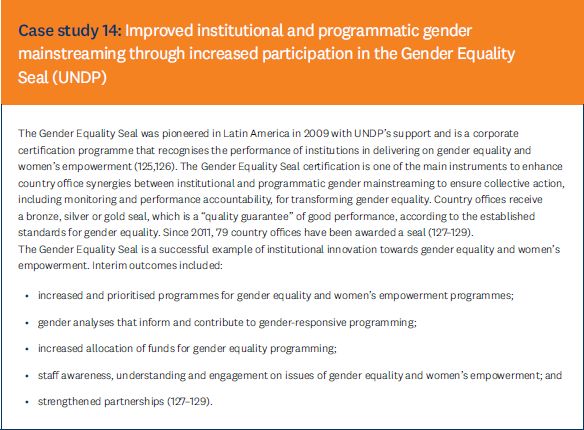

Supplement: S1 Text — (DOCX) [file pgph.0004697.s001.docx]
