# Supplementary material for: Advancing gender equality in global health: What can we learn from successful gender integration across five UN agencies?
Source: PLOS Glob Public Health. 2025 Jun 4;5(6):e0004697. doi: 10.1371/journal.pgph.0004697 (PMC12136305; doi:10.1371/journal.pgph.0004697)
Supplement: Attachment — Submitted filename: Gender-Review_31122024.pdf [file pgph.0004697.s002.pdf]

PGPH-D-24-01960

Advancing Gender Equality in Global Health: What Can We Learn From Successful Gender Integration Across Five UN Agencies?

PLOS Global Public Health

S. No.	Criteria	Review Comments
**1. General Comments**		
1.1	1.1.1.1 What is the study about?<br><br>1.1.2. What is the main research question?	1.1.1. There is inconsistency between 'Abstract' & 'Introduction' in explaining the study topic and its rationale:<br>As per the abstract, "the global on-going crises (climate, economic, social, political – and their impact on health) have worsened health inequities and eroded strides advancing gender equality, with detrimental impacts in the health sector and beyond". (Lines 28-33)<br>However, the introduction is not reiterating the argument that global ongoing crises are hindering advancement towards gender equality. Rather it mentions that the "impact of on-going global crises has raised the urgency to keep gender equality on the global health agenda and for leaders to take corrective action". (Lines 52-55)<br>The authors need to be consistent in their argument and align abstract with introduction.<br><br>1.1.2. As a result of confusion in the study topic, the research aim, and question also remain unclear.
1.2.	1.2.1. What is the approach?<br><br>1.2.2. What did the authors do to address their research question?	1.2.1. The authors mentioned that "Data collection was informed by the underlying principles of Critical Realist Approach" (Line-100).<br>However, it is unclear whether this approach was used only for data collection or for analysis as well.<br>1.2.2. The authors mentioned that they reviewed and analyzed 14 case studies from five UN agencies. However, the framework or template used for analysis is not mentioned. Autheros should add the analysis framework.

1.3	1.3.1. What is the context?<br><br>1.3.2. How does the study relate to published literature on this topic?	1.3.1. The context is mainly UN agencies/organizations. The bigger context like programs other than Un, other countries or regions, are not mentioned in the Introduction. There is very little information on the context of this study.<br>1.3.2. The literature review on the study topic is extremely inadequate. Only references are givn in the introduction about the context.
1.4	1.4.1. What are the conclusions?<br>1.4.2. What are the authors' main findings and what evidence do they provide for these conclusions?	1.4.1. Conclusions are not given separately. In the discussion section, the authors have mentioned 5 key success ingredients for integrating gender for gender equality. (Line 517-518).<br>1.4.2. It is unclear if the framework or templates for analyzing and assessing the 'Gender Integration' and 'Gender Equality' are not mentioned, how to validate study methods and findings.
**2. Abstract & Introduction**		
2.1	2.1. Overall context and approach	2.1. The manuscript mainly focuses on UN agencies/organizations context.<br>A 'Critical Realist Approach' is being used (Line -100). Inadequate literature review of basic concepts, measures and outcomes.
2.2	Do the authors summarize the main research question and key findings?	2.2. The abstract describes that the study was done to identify what has worked institutionally and programmatically to promote gender equality in health over the last 25 years. However, the existing scientific knowledge has not been presented. Because of this inadequacy, it is difficult to assess the research gap and how would this study fill this gap.<br>The key findings are given under discussion.
2.3	2.3. Do the authors identify other literature on the topic and explain how the study relates to this previously published research?	2.3.1. There are only 4 references in the 'Introduction'.<br>2.3.2. The introduction should have covered the following key areas:<br>i. How does literature define 'Gender Equality', 'Women's Empowerment' and 'Gender Integration'?<br>ii. What is the authors' perspective on the definitions of 'Gender Equality', 'Women's Empowerment' and 'Gender Integration'?<br>iii. What operational definitions have authors used for defining 'Gender Equality', 'Women's Empowerment' and 'Gender Integration'?<br>iv. Why 'Gender Equality' is important and how it contributes to 'women Empowerment'?<br>v. What is 'Gender Integration' and how is it linked with 'Gender Equality' and 'Women Empowerment'?<br>2.3.3. Most part of the 'Introduction' is describing the UN system and its strengths. (Line: 61-70).

		2.3.4. The research gap is basically identified from UN-based work. Literature other than UN agencies should have been mentioned. (lines: 79-82). The authors need to present scientific literature from other settings to mention what has been done on this topic so far and then identify the research gap.<br>2.3.5. The aims are focusing on 'Successful programmatic & institutional gender integration', 'determinants & outcomes of gender integration' and 'commonalities and lessons across success cases' (lines: 84-89). However, the definitions and standards of these key words are not given in the methods.<br>2.3.6. It is also not mentioned which framework is used to assess the case study outcome and success.<br>2.37. Nothing has been mentioned about what has been done so far in scientific research on these aspects and domains.
**3. Figures & Tables**		
3.1.	3.1. Are the figures and tables clear and readable?	3.1. Most of the tables/Figures are clear and readable except the last one.
3.2.	3.2. Are the figure and table captions complete and accurate?	3.2. The figure and table captions are complete and accurate
3.3.	3.3. Are the axes labeled correctly?	3.3. The axes are labeled correctly
3.4.	3.4. Is the presentation appropriate for the type of data being presented?<br>Do the figures and tables support the findings?	3.4. The figures and tables support the findings.
**4. Methods**		
4.1.	4.1. Are the experiments or interventions appropriate for addressing the research question?	4.1.1. Methods have comprehensively described all the steps and processes of the study.<br>4.1.2. However, the research is not technically sound.<br>i. The missing area is the description of research design and methods. It is not clear from the methods section which research design, and techniques were used.<br>ii. The authors need to mention the scientific research design and methods used and their 'Why & How'.

		ii.Justification needs to be added for the selected research design and methods.
4.2.	4.2. Are conditions adequate and the right controls in place?	4.2. The major deficiency is scientific rigor.
4.3.	4.3. Is there enough data to draw a conclusion?	4.3. Data was enough but not analyzed using standard frameworks.
4.4.	4.4. Do the authors address any possible limitations of the research?	4.4. Limitations need to be added
4.5.	4.5. Was data collected and interpreted accurately?	4.5. This study utilized secondary data. Since the research design and methods used and their justifications are not given, it is difficult to comment on their appropriateness of interpretation.
4.6.	4.6. Do the authors follow best practices for reporting?	4.6. The analysis is not based on scientific methods and standard templates/frameworks are not used.
4.7.	4.7. What experiments or interventions were used?	4.7. The authors reviewed documents, conducted Key Informant Interviews and workshops. But it is not clear why these techniques were selected. And what guidelines were used for interviews and workshops.
4.8.	4.8. Does the study conform to ethical guidelines?	4.8. Authors need to clearly describe this important aspect.
4.9.	4.9. Could another researcher reproduce the study with the same methods?<br>In other words, have the authors provided enough information to validate the study?	4.9.1. Another researcher could reproduce the study following the steps and processes mentioned in the methods section.<br>4.9.2. However, scientific rigor is missing in the manuscript.<br>i. The standard frameworks used for analysis are not mentioned.<br>ii. The research design and data collection techniques and the justification for selecting those are not given.<br>iii. What was the sample size and how was that calculated?<br>iv. What were the sampling criteria?<br>v. How were participants selected?<br>vi. Whether interviews were conducted using any guide? If yes, how was that guide developed?
**5. Results, Discussion & Conclusions**		
5.1.	5.1. Do the results support the conclusions?	There is no separate section for conclusion. Some key findings are given in the discussion.

5.2.	5.2. Do the conclusions overreach?	5.2. Since standard analytical frameworks are not given, it is difficult to comment on the findings and conclusions.
5.3.	5.3. Do the authors discuss any limitations of the study?	No
5.4.	5.4. If the journal selects based on advance in the field does the study demonstrate this advance?	Scientific rigor is missing
**6. Statistical Analysis**		
6.1.	6.1. Is the statistical analysis adequate? If you do not have the expertise to consider the statistics, make sure you mention this in your report.	6.1. Not Applicable
6.2.	6.2. Data and supporting information	6.2. Data is available
6.3.	6.3. Do the data provide enough evidence for the authors' conclusions?	6.3. Data provides evidence. However, the standards for comparing data are not given.
6.4.	6.4. Are the necessary data points provided?	6.4. Data is presented comprehensively
6.5.	Have the authors provided a sufficient amount of data and information for other researchers to recreate the analyses?	6.5. The analytical standards and frameworks are not presented.
**7. Writing Quality & Clarity**		

7.1.	7.1. As a reviewer, you should focus on the substance of the research rather than the writing. If you think the quality of the writing needs to be improved, don't spend your time pointing out individual typos and other minor details. Just mention in your comments that you recommend language editing.	7.1. The quality of writing is extremely good.
