# Supplementary material for: Advancing gender equality in global health: What can we learn from successful gender integration across five UN agencies?
Source: PLOS Glob Public Health. 2025 Jun 4;5(6):e0004697. doi: 10.1371/journal.pgph.0004697 (PMC12136305; doi:10.1371/journal.pgph.0004697)
Supplement: Attachment — Submitted filename: Gender-Review_31122024_responses_answered.docx [file pgph.0004697.s004.docx]

PGPH-D-24-01960

Advancing Gender Equality in Global Health: What Can We Learn From Successful Gender Integration Across Five UN Agencies?

PLOS Global Public Health

| **Editor’s Comments** | **Response from authors** |
| --- | --- |
| 1. Clarification of Interview Data Usage: In the methodology section, the authors mention conducting interviews with key informants from several UN agencies. However, there is no clear analysis of these interviews in the findings. If interview data contributed to the results, the authors should specify which sections were informed by the interviews, supported by relevant quotes. If not, references to the interviews should be removed to avoid confusion. | We thank the editor for their comment. To clarify, the key informant interviews provided individual perspectives on 1) what were the agency successes towards gender equality in health programmes and institutionally, and 2) what factors contributed to the successes. These initial data were then augmented through evidence from further web searches and organisation-specific virtual workshops. The process was iterative, with each level adding to and refining the information previously gathered. Ultimately the goal was to create a context-mechanisms-outcomes framework, informed by a critical realist approach, for each case study so as to identify the drivers of success (which is one of the study objectives). Therefore, providing individual quotes from the interviews does not make sense given the consolidated and iterative nature of the analysis and the ultimate goal of generating the context-mechanisms-outcomes framework.  To clarify this further we have amended the text in the methodology section as follows:  *“Once case-study-specific information was validated through agency-specific workshops, analyses were conducted iteratively, evolving and building on new insights and emerging patterns. To do this, we reviewed interview transcripts once again, drew on the background reviews and carried out further web-based research to identify the contextual factors and potential mechanisms for each successful outcome. These sources included internal reports available on agency websites, such as reports to the executive committee, and reports of independent evaluations. This was supplemented by internal documents that the agencies were able to make available. A detailed scrutiny of the information on each successful case study led to the development of a context-mechanisms-outcome framework for analysing the constellation of factors driving successful gender integration initiatives, which was underpinned by the critical realist approach described above.”* (Lines 223 – 233) |
| 2. Reflexivity and Positionality: Given that the study was conducted within an institution closely linked to the UN, the authors should include a reflexivity statement. This should clarify how their professional and educational backgrounds, as well as institutional ties with UN agencies, may have influenced the research process, analysis, and interpretation of findings. | We have taken on board this helpful feedback and included a section prior to results where we reflect on our positionality as a UN institute:  *“Positionality of the institute and the research team*  *UNU-IIGH is an independent global health think tank within the UN system with a mandate to advance the perspectives and needs of states and populations in the Global South to catalyse equitable shifts in power to advance global health. UNU-IIGH works with experts, practitioners, policy-makers and academics, including UN agencies. Throughout these interactions, however, it maintains its role as a neutral convener with its staff and associates maintaining their academic independence. This positionality allows us to critically reflect on the UN system’s response to gender integration in health and ensure objectivity and impartial analysis of the evidence collected.”* (Lines 258 – 266) |
| 3. Enhancing the Discussion Section: The first part of the discussion largely reiterates findings without deeper analysis. While summarizing key results is useful, this section should critically explore the implications of the findings. For example, while highlighting successes within UN agencies, the authors could contrast these with independent academic evaluations of the real-world impact on women’s lives. Integrating relevant published literature will strengthen this analysis. Additionally, the discussion should explicitly acknowledge the study’s limitations, providing a foundation for future research. | We appreciate the editors comments however to our knowledge there are no other studies that have examined *why* particular interventions that integrate gender in health programmes were successful. Of the evidence base that does exist, it focuses predominantly on evaluating *whether* a programme was successful rather than the practice-based realities of *how* success came about. This is what is so unique about our approach and the significant contribution our study makes to the evidence base to advance gender integration in health programmes.  To further clarify this point we have added the following text to our discussion and also included a section on the study limitations:  *“In terms of drawing on published literature to reflect critically on the successes mentioned, to our knowledge, this is the first study of its kind to analyse factors that contributed to successful cases of gender integration in health, programmatically or institutionally, across multiple UN agencies. Where evidence has been published, it is often based on independent evaluations of singular programmes and assessed whether a programme was successful rather than how or what enabled it to be successful. This unique feature of this study therefore sets it apart from the evidence base that exists of formal evaluations of UN programmes focusing on gender integration in health.”* (Lines 646 – 653).  *“3.5 What were the limitations of this study*  *One of the study’s limitations was that it was based on a review of documents, and a limited number of virtual key informant interviews, followed by a virtual analysis workshop. Because of resource, time, and physical constraints due to COVID-19, we could not include gender focal points experts from multiple country offices or any of the partners involved in programme implementation or programme beneficiaries at the country level as key informants. Thus, our information on successes is limited to what was widely perceived to be successes at the headquarters and regional office-level. Also, the scope of the study did not extend to examining the successful outcomes in gender integration in health within the contexts of specific countries and through specific implementing partners. The study also does not analyse what did not work and why, which could serve as a valuable comparison to the successful case studies analysed.”* (Lines 581 - 591) |
| 4. Clarification of Ethics Approval: Although the study is led by the United Nations University International Institute for Global Health, ethical approval was sought from Monash University. This discrepancy requires clarification to ensure transparency in the research process. | The PI for the study, Prof Pascale Allotey, was originally at Monash University and subsequently took over the Directorship at UNU-IIGH. Ethics approval had been obtained from Monash prior to this move. |
| 5. Declaration of Conflicts of Interest: The authors should explicitly declare any potential conflicts of interest to maintain the integrity of the publication process. I hope these suggestions help improve the manuscript’s rigor and critical engagement with the topic. | The authors declare no conflicts of interest. |

| **S. No.** | **Criteria** | **Review Comments** | **Response from authors** |
| --- | --- | --- | --- |
| **1. General Comments** | | |  |
| 1.1 | 1.1.1.1 What is the study about?  1.1.2. What is the main research question? | 1.1.1. There is inconsistency between ‘Abstract’ & ‘Introduction’ in explaining the study topic and its rationale:   As per the abstract, “the global on-going crises (climate, economic, social, political – and their impact on health) have worsened health inequities and eroded strides advancing gender equality, with detrimental impacts in the health sector and beyond”. (Lines 28-33)  However, the introduction is not reiterating the argument that global ongoing crises are hindering advancement towards gender equality. Rather it mentions that the “impact of on- going global crises has raised the urgency to keep gender equality on the global health agenda and for leaders to take corrective action”. (Lines 52-55)  The authors need to be consistent in their argument and align abstract with introduction.   1.1.2. As a result of confusion in the study topic, the research aim, and question also remain unclear. | 1.1.1.1 Thank you for this point. We have added in a further explanation in the introduction to clarify that on-going global crises are, *“having detrimental effects on advances in health equity and gender equality.”* (Lines 65-66).  1.1.2 The authors do not agree that the study topic, aims and research questions are not clear. Please see lines 97-108 where the study topic, aims and objectives are clearly stated. |
| 1.2. | 1.2.1. What is the approach? 1.2.2. What did the authors do to address their research question? | 1.2.1. The authors mentioned that “Data collection was informed by the underlying principles of   Critical Realist Approach” (Line-100).  However, it is unclear whether this approach was used only for data collection or for analysis as well.   1.2.2. The authors mentioned that they reviewed and analyzed 14 case studies from five UN agencies. However, the framework or template used for analysis is not mentioned. Authors should add the analysis framework. | 1.2.1 We thank the reviewer for this feedback. We have included further clarification that the critical realist approach was used for analyses as well as data collection and justified why a realist approach was best suited for this study:  *“Realist synthesis is a theory-driven approach that sets out to understand “what works, where, for whom, why and how” rather than simply evaluate if an intervention works (68). A realist approach was considered well-suited for this study for several reasons: first, because this study aimed to develop a detailed understanding of the features that led to successful gender integration; second, due to the limited understanding of the pathways leading to successful outcomes in gender integration which is taken into consideration in a realist approach; and third, because of the complexity of social realities that encompass the cases which a realist approach accounts for (68, 69). Underpinning the realist approach is the underlying principle that particular mechanisms, actions, and associated changes lead to desired outcomes within specific contexts (68).*  *Data collection and analyses were therefore as informed by the underlying principle of a critical realist approach, that the relationship between contexts, mechanisms and outcomes needs to be explored to understand how and why an outcome was achieved in some contexts but not others (11).”* (Lines 120 - 134).  1.2.2 We have taken on board the feedback and included the following paragraph detailing how analyses were conducted.  *“Four sequential steps, detailed below, were then used to identify and select case studies and gather and analyse relevant primary and secondary data for the study based on the context, mechanisms and outcomes framework underpinning the realise approach used.”* (Lines 145 – 148)  *“Step three involved the development of the context-mechanisms-outcome framework and analysis of findings. Once case-study-specific information was validated through agency-specific workshops, analyses were conducted iteratively, evolving and building on new insights and emerging patterns. To do this, we reviewed interview transcripts once again, drew on the background reviews and carried out further web-based research to identify the contextual factors and potential mechanisms for each successful outcome. These sources included internal reports available on agency websites, such as reports to the executive committee, and reports of independent evaluations. This was supplemented by internal documents that the agencies were able to make available. A detailed scrutiny of the information on each successful case study led to the development of a context-mechanisms-outcome framework for analysing the constellation of factors driving successful gender integration initiatives, which was underpinned by the critical realist approach described above.”* (Line 222 – 233) |

| 1.3 | 1.3.1. What is the context? 1.3.2. How does the study relate to published literature on this topic? | 1.3.1. The context is mainly UN agencies/organizations. The bigger context like programs other than Un, other countries or regions, are not mentioned in the Introduction. There is very little information on the context of this study. 1.3.2. The literature review on the study topic is extremely inadequate. Only references are givn in the introduction about the context. | 1.3.1 and 1.3.2 We thank the reviewer for raising this point. We have taken on board this feedback and added in an introductory paragraph situating more of the context in terms of existing evidence that gender inequalities and restrictive norms adversely affect health but also the major gap in practice based evidence of what works to address gender inequalities in health programmes and institutions, especially from the perspective of multilateral institutions that play a considerable role in advancing gender equality in health programmes globally.  *“The universal right to “the enjoyment of the highest attainable standard of health is one of the fundamental rights of every human being without distinction of race, religion, political belief, economic or social condition (64).” Meeting this goal however requires addressing harmful inequalities in power at a social level – be they control over resources, decision-making, access to services, information, and opportunities, all of which impact health and wellbeing. Extensive evidence exists showcasing that one of the primary axes along which harmful power is wielded are socially constructed gendered roles, responsibilities and norms, features that health programmes and institutions often reproduce (65 - 67). Far less researched, however, is what works in practice to address these gendered inequalities especially from the perspective of major multilateral actors that work extensively on advancing health equity and gender equality.”* (Lines 52-61) |
| --- | --- | --- | --- |
| 1.4 | 1.4.1. What are the conclusions? 1.4.2. What are the authors’   main findings and what  evidence do they provide for these conclusions? | 1.4.1. Conclusions are not given separately. In the discussion section, the authors have mentioned 5 key success ingredients for integrating gender for gender equality. (Line 517-518). 1.4.2. It is unclear if the framework or templates for analyzing and assessing the ‘Gender Integration’ and ‘Gender Equality’ are not mentioned, how to validate study methods and findings. | 1.4.1 and 1.4.2 Given the study objectives were as follows:   A. Document the types of outcomes UN agencies have been able to achieve in terms of successful programmatic and institutional gender integration in health; B. Identify the determinants that led to the outcomes in successful cases of programmatic and institutional gender integration in health; and C. Distil commonalities and lessons across successful cases to constructively inform future work on gender integration within the UN system and other bilateral and multilateral organisations working in health.   The main findings that speak to each of these objectives are presented and substantiated with evidence in the following sections:   A. See section 3.1 (Lines 272 – 311) B. See sections 3.2 - 3.4 (Lines 313 – 519) C. See section 4 (Lines 593 – 726)   Although not labeled explicitly as ‘conclusions’ section 4.2 Way Forward (Lines 697 – 726) presents the overall conclusions based on the evidence gathered, analysed and presented in prior sections. |
| **2. Abstract & Introduction** | | |  |
| 2.1 | 2.1. Overall context and approach | 2.1. The manuscript mainly focuses on UN agencies/organizations context.  A ‘Critical Realist Approach’ is being used (Line -100). Inadequate literature review of basic concepts, measures and outcomes. | 2.1 Yes, the primary focus on this research project was five UN agencies, therefore the introduction largely speaks specifically to the UN context and all subsequent sections related to key findings.  We have expanded the methodology section to detail how data were analysed in relation to the critical realist approach (see earlier response to 1.2.2). |
| 2.2 | Do the authors summarize the main research question and key findings? | 2.2. The abstract describes that the study was done to identify what has worked institutionally and programmatically to promote gender equality in health over the last 25 years. However, the existing scientific knowledge has not been presented. Because of this inadequacy, it is difficult to assess the research gap and how would this study fill this gap.  The key findings are given under discussion. | 2.2 Please see our response to 1.3.1 and 1.3.2 where we have taken on board this feedback and included an additional paragraph in the introduction explaining the existing evidence but also the gaps specifically in relation to practice-based evidence from multilateral institutions who play considerable roles in advancing gender equality in health programmes globally. |
| 2.3 | 2.3. Do the authors identify other literature on the topic and explain how the study relates to this previously published research? | 2.3.1. There are only 4 references in the ‘Introduction’. 2.3.2. The introduction should have covered the following key areas:        i. How does literature define ‘Gender Equality’, ‘Women’s Empowerment’ and ‘Gender Integration’?        ii. What is the authors’ perspective on the definitions of ‘Gender Equality’, ‘Women’s Empowerment’ and ‘Gender Integration’?        iii. What operational definitions have authors used for defining ‘Gender Equality’, ‘Women’s Empowerment’ and ‘Gender Integration’?        iv. Why ‘Gender Equality’ is important and how it contributes to ‘women Empowerment’?        v. What is ‘Gender Integration’ and how is it linked with ‘Gender Equality’ and ‘Women Empowerment’? 2.3.3. Most part of the ‘Introduction’ is describing the UN system and its strengths. (Line: 61-70). | 2.3.1 - 2.3.4 Please see our response to 1.3.1 and 1.3.2. We have taken on board this feedback and added in an introductory paragraph (Lines 52-61) situating more of the context in terms of existing evidence that gender inequalities and restrictive norms adversely affect health but also the major gap in practice based evidence of what works to address gender inequalities in health programmes and institutions, especially from the perspective of multilateral institutions that play a considerable role in advancing gender equality in health programmes globally.  Given the aim of this paper was what we can learn from cases of what has worked institutionally and programmatically to promote gender equality in health within five UN agencies, we intentionally chose not to focus on gender language, definitions, and operationalization, as this is an entirely different research question. |

|  |  | 2.3.4. The research gap is basically identified from UN-based work. Literature other than UN agencies should have been mentioned. (lines: 79-82). The authors need to present scientific literature from other settings to mention what has been done on this topic so far and then identify the research gap. 2.3.5. The aims are focusing on ‘Successful programmatic & institutional gender integration’, ‘determinants & outcomes of gender integration’ and ‘commonalities and lessons across success cases’ (lines: 84-89). However, the definitions and standards of these key words are not given in the methods. 2.3.6. It is also not mentioned which framework is used to assess the case study outcome and success.   2.37. Nothing has been mentioned about what has been done so far in scientific research on these aspects and domains. | 2.3.5 and 2.3.6 We thank the reviewers for this point and have expanded the definition of success as follows to give further clarity:  *“Successful cases were defined as follows:*   1. *Programmatic gender integration – health programmes that resulted in positive gender equality and health outcomes sustained for at least five years. Criteria for defining improvements of health outcomes related to addressing women’s, men’s and/or gender-diverse people’s specific health needs and gender equality outcomes related to altering unequal power relations that are detrimental to health.* 2. *Institutional gender integration – internal organisational changes that demonstrated improved gender equality, specifically unequal power relations, at the organisational level or significantly enabled programmatic gender integration in health.”* (Lines 192 – 200)   2.3.7 Please see our earlier point above regarding the focus on this work. We did not set out to assess definitions and operationalisation of success with regards to gender equality and health. We set out to understand, given successful cases that the agencies themselves identified, *how* the successes came about. This is the piece that is widely missing in the existing literature. |
| --- | --- | --- | --- |
| **3. Figures & Tables** | | |  |
| 3.1. | 3.1. Are the figures and tables clear and readable? | 3.1. Most of the tables/Figures are clear and readable except the last one. | 3.1 We have updated the last figure to be a higher resolution. |
| 3.2. | 3.2. Are the figure and table captions complete and  accurate? | 3.2. The figure and table captions are complete and accurate |  |
| 3.3. | 3.3. Are the axes labeled correctly? | 3.3. The axes are labeled correctly |  |
| 3.4. | 3.4. Is the presentation appropriate for the type of data being presented?  Do the figures and tables  support the findings? | 3.4. The figures and tables support the findings. |  |
| **4. Methods** | | |  |
| 4.1. | 4.1. Are the experiments or interventions appropriate for addressing the research question? | 4.1.1. Methods have comprehensively described all the steps and processes of the study. 4.1.2. However, the research is not technically sound.  i. The missing area is the description of research design and methods. It is not clear from the methods section which research design, and techniques were used. ii. The authors need to mention the scientific research design and methods used and their ‘Why &   How’. | 4.1.1 - 4.1.2 We have taken on board this feedback and included a longer justification about why a realist approach was appropriate and selected for this study. The following text has now been included:  *“Realist synthesis is a theory-driven approach that sets out to understand “what works, where, for whom, why and how” rather than simply evaluate if an intervention works (68). A realist approach was considered well-suited for this study for several reasons: first, because this study aimed to develop a detailed understanding of the features that led to successful gender integration; second, due to the limited understanding of the pathways leading to successful outcomes in gender integration which is taken into consideration in a realist approach; and third, because of the complexity of social realities that encompass the cases which a realist approach accounts for (68, 69). Underpinning the realist approach is the underlying principle that particular mechanisms, actions, and associated changes lead to desired outcomes within specific contexts (68).*  *Data collection and analyses were therefore as informed by the underlying principle of a critical realist approach, that the relationship between contexts, mechanisms and outcomes needs to be explored to understand how and why an outcome was achieved in some contexts but not others (11).”* (Lines 120 – 134) |

|  |  | ii.Justification needs to be added for the selected research design and methods. |  |
| --- | --- | --- | --- |
| 4.2. | 4.2. Are conditions adequate and the right controls in place? | 4.2. The major deficiency is scientific rigor. | 4.2 We disagree with this statement. The study is firmly grounded in a critical realist approach and was carefully designed and implemented to ensure the upmost scientific rigor. The unique nature of drawing lessons from practice-based evidence from within the UN, which is a major gap in the existing literature, means dealing with social complexities, a feature that realist approaches allow for. |
| 4.3. | 4.3. Is there enough data to draw a conclusion? | 4.3. Data was enough but not analyzed using standard frameworks. | 4.3 As stated in the updated methodology section, the analysis was informed by a critical realist approach which meant that data were analysed based on: 1. types of outcomes achieved, 2. contextual factors present and 3. actors and actions (mechanisms) which triggered and sustained change.  The findings section clearly presents analysed data based on these three areas which align directly to the theoretical framework presented and justified according to the study aims and objectives. |
| 4.4. | 4.4. Do the authors address any possible limitations of  the research? | 4.4. Limitations need to be added | 4.4. We appreciate this useful feedback and have added a limitations section.  “*3.5 What were the limitations of this study*  *One of the study’s limitations was that it was based on a review of documents, and a limited number of virtual key informant interviews, followed by a virtual analysis workshop. Because of resource, time, and physical constraints due to COVID-19, we could not include gender focal points experts from multiple country offices or any of the partners involved in programme implementation or programme beneficiaries at the country level as key informants. Thus, our information on successes is limited to what was widely perceived to be successes at the headquarters and regional office-level. Also, the scope of the study did not extend to examining the successful outcomes in gender integration in health within the contexts of specific countries and through specific implementing partners. The study also does not analyse what did not work and why, which could serve as a valuable comparison to the successful case studies analysed.”* (Lines 581 – 591) |
| 4.5. | 4.5. Was data collected and interpreted accurately? | 4.5. This study utilized secondary data. Since the research design and methods used and their justifications are not given, it is difficult to comment on their appropriateness of interpretation. | 4.5 The study utilized both primary and secondary data (see Lines 145 – 256) to develop the outcomes, context and mechanisms framework based on the realist approach.  To further clarify this, we have added the following text in the methodology section:  *“Four sequential steps, detailed below, were then used to identify and select case studies and gather and analyse relevant primary and secondary data for the study based on the context, mechanisms and outcomes framework underpinning the realise approach used.”* (Lines 145 – 148) |
| 4.6. | 4.6. Do the authors follow best practices for reporting? | 4.6. The analysis is not based on scientific methods and standard templates/frameworks are not used. | 4.6 To further clarify how the analysis is deeply embedded in scientific frameworks (critical realist approach) we have added in the following text:  *“Step three involved the development of the context-mechanisms-outcome framework and analysis of findings. Once case-study-specific information was validated through agency-specific workshops, analyses were conducted iteratively, evolving and building on new insights and emerging patterns. To do this, we reviewed interview transcripts once again, drew on the background reviews and carried out further web-based research to identify the contextual factors and potential mechanisms for each successful outcome. These sources included internal reports available on agency websites, such as reports to the executive committee, and reports of independent evaluations. This was supplemented by internal documents that the agencies were able to make available. A detailed scrutiny of the information on each successful case study led to the development of a context-mechanisms-outcome framework for analysing the constellation of factors driving successful gender integration initiatives, which was underpinned by the critical realist approach described above.”* (Line 222 – 233) |
| 4.7. | 4.7. What experiments or interventions were used? | 4.7. The authors reviewed documents, conducted Key Informant Interviews and workshops.  But it is not clear why these techniques were selected. And what guidelines were used for interviews and workshops. | 4.7 We have taken on board this feedback and added the following sentence to clarify the purpose of the key informant interviews:  *“The purpose of the key informant interviews was to identify successes in gender integration in health, institutionally and programmatically, drawing out explanations on the contextual factors and mechanisms behind sustained successes.”* (Lines 160 – 163) |
| 4.8. | 4.8. Does the study conform to ethical guidelines? | 4.8. Authors need to clearly describe this important aspect. | 4.8 Please see the following sentence where we specify that we adhered to ethical standards:  *“All interview material, audio, video and transcripts were stored securely, adhering to our research and United Nations Evaluation Group ethical standards (12-13).”* (Lines 185 – 187) |
| 4.9. | 4.9. Could another researcher reproduce the study with the same methods?  In other words, have the authors provided enough information to validate the study? | 4.9.1. Another researcher could reproduce the study following the steps and processes mentioned in the methods section. 4.9.2. However, scientific rigor is missing in the manuscript.  i. The standard frameworks used for analysis are not mentioned. ii. The research design and data collection techniques and the justification for selecting those are not given. iii. What was the sample size and how was that calculated? iv. What were the sampling criteria? v. How were participants selected? vi. Whether interviews were conducted using any guide? If yes, how was that guide developed? | 4.9 The study is firmly grounded in a critical realist approach and was carefully designed and implemented to ensure the upmost scientific rigor. As described above (responses 1.2.1, 1.2.2 ), we have amended the methods section to more clearly delineate the scientific rigor with which the study was conducted.  In addition, we have added the following text to explain how the sample size was calculated for the key informant interviews and the sampling criteria used:  *“The sampling method was purposeful, informed and guided by the agency focal point in consultation with the study team, aiming to collect perspectives from a breath of relevant agency stakeholders at headquarter and regional level. The sample size was determined based on reaching saturation of successful cases identified, and the time, resource and travel constraints due to COVID-19.*  *Two retired UN officials, who had held senior management positions and had extensive expertise in gender equality and women’s empowerment, conducted the interviews based on an interview guide co-developed with the study team and the agency focal points.”* (Line 175 – 183) |
| **5. Results, Discussion & Conclusions** | | |  |
| 5.1. | 5.1. Do the results support the conclusions? | There is no separate section for conclusion. Some key findings are given in the discussion. | 5.1 Given the study objectives were as follows:  A. Document the types of outcomes UN agencies have been able to achieve in terms of successful programmatic and institutional gender integration in health; B. Identify the determinants that led to the outcomes in successful cases of programmatic and institutional gender integration in health; and C. Distil commonalities and lessons across successful cases to constructively inform future work on gender integration within the UN system and other bilateral and multilateral organisations working in health.   The main findings that speak to each of these objectives are presented and substantiated with evidence in the following sections:   A. See section 3.1 (Lines 272 – 311) B. See sections 3.2 - 3.4 (Lines 313 – 519) C. See section 4 (Lines 593 – 726) |

| 5.2. | 5.2. Do the conclusions overreach? | 5.2. Since standard analytical frameworks are not given, it is difficult to comment on the findings and conclusions. | 5.2 We have provided further clarify and justification in the methodology with regards to the analytical framework used.  In terms of the conclusions, although not labeled explicitly as ‘conclusions’ section 4.2 Way Forward (Lines 697 – 726) presents the overall conclusions based on the evidence gathered, analysed and presented in prior sections. |
| --- | --- | --- | --- |
| 5.3. | 5.3. Do the authors discuss any limitations of the study? | No | 5.3 We have since included a section discussing limitations:   “*3.5 What were the limitations of this study*  *One of the study’s limitations was that it was based on a review of documents, and a limited number of virtual key informant interviews, followed by a virtual analysis workshop. Because of resource, time, and physical constraints due to COVID-19, we could not include gender focal points experts from multiple country offices or any of the partners involved in programme implementation or programme beneficiaries at the country level as key informants. Thus, our information on successes is limited to what was widely perceived to be successes at the headquarters and regional office-level. Also, the scope of the study did not extend to examining the successful outcomes in gender integration in health within the contexts of specific countries and through specific implementing partners. The study also does not analyse what did not work and why, which could serve as a valuable comparison to the successful case studies analysed.”* (Lines 581 – 591) |
| 5.4. | 5.4. If the journal selects based on advance in the field does the study  demonstrate this advance? | Scientific rigor is missing | 5.4 As stated earlier, we disagree that the study lacks scientific rigor. The study is firmly grounded in a critical realist approach and was carefully designed and implemented to ensure the upmost scientific rigor. The unique nature of drawing lessons from practice-based evidence from within the UN, which is a major gap in the existing literature, means dealing with social complexities, a feature that realist approaches allow for. |
| **6. Statistical Analysis** | | |  |
| 6.1. | 6.1. Is the statistical analysis adequate? If you do not have the expertise to consider the statistics, make sure you mention this  in your report. | 6.1. Not Applicable |  |
| 6.2. | 6.2. Data and supporting information | 6.2. Data is available |  |
| 6.3. | 6.3. Do the data provide enough evidence for the  authors’ conclusions? | 6.3. Data provides evidence. However, the standards for comparing data are not given. |  |
| 6.4. | 6.4. Are the necessary data points provided? | 6.4. Data is presented comprehensively |  |
| 6.5. | Have the authors provided a sufficient amount of data and information for other researchers to recreate the  analyses? | 6.5. The analytical standards and frameworks are not presented. |  |
| **7. Writing Quality & Clarity** | | |  |

| 7.1. | 7.1. As a reviewer, you should focus on the substance of the research rather than the writing. If you think the quality of the writing needs to be improved, don’t spend your time pointing out individual typos and other minor details. Just mention in your comments that you recommend language  editing. | 7.1. The quality of writing is extremely good. |  |
| --- | --- | --- | --- |
